# Neural manifestation of L2 novel concept acquisition from multi-contexts via both episodic memory and semantic memory systems

**DOI:** 10.3389/fpsyg.2024.1320675

**Published:** 2024-02-07

**Authors:** Shuang Xu, Hailing Wang, Shouxin Li, Guang Ouyang

**Affiliations:** ^1^Faculty of Education, University of Hong Kong, Pokfulam, Hong Kong SAR, China; ^2^School of Psychology, Shandong Normal University, Jinan, China

**Keywords:** word meaning, N400, semantic network, L2 acquisition, contextual learning

## Abstract

This study aims to examine the process of L2 novel word learning through the combination of episodic and semantic memory, and how the process differs between the formation of thematic and taxonomic relations. The major approach adopted was observing the neural effects of word learning, which is manifested in the N400 from event-related potentials (ERPs). Eighty-eight participants were recruited for the experiment. In the learning session, L2 contextual discourses related to novel words were learned by participants. In the testing session, discourses embedded with incongruous and congruous novel words in the final position were used for participants to judge the congruency which affected the N400 neural activity. The results showed that both recurrent and new-theme discourses elicited significant N400 effects, while taxonomic sentences did not. These results confirmed the formation of episodic and semantic memory during L2 new word learning, in which semantic memory was mainly supported by thematic relations.

## Introduction

In the language evolution in human society, there is the constant appearance of new concepts and the disappearance of old ones. Concepts are conveyed by words. A word can be seen as being constituted of two parts: form and meaning. In this sense, successful learning of a new word can be described as the process of building the link between word form and meaning, and the learning of new meaning is realized by linking it to an existing semantic network, thus forming a larger network (Nematzadeh et al., 2014). In the present work that studies the learning of word meaning, we focused specifically on lexical semantics which refers to the meaning contents represented by word forms (Geeraerts, 2009). At the individual level, the development and establishment of the conceptual semantic network in the brain is a life-long process in which new concepts are constantly being established and existing concepts are extended and renewed, and consequently, the connectivity patterns among concepts are constantly evolving (Nelson et al., 2013; Sowa, 2014; Kennedy et al., 2015). Given the general process of word learning across the lifespan, it would be interesting and important to know how this process is manifested in learning of second language (termed L2 learning later), which would be presumably more complicated than the natural process of learning first language (termed L1 learning later).

Among many ways through which people acquire novel concepts in L2, contextual learning plays a crucial role, especially in word learning (Nassaji, 2003; Elgort et al., 2018). People often actively infer word meaning from the contextual information in the discourses in which the word is embedded, rather than being passively taught the meaning of words (Mestres-Missé et al., 2008, 2009). Effective contextual learning relies on abundant and high-constraint discourses through which novel words are linked with known words (Batterink and Neville, 2011). To investigate these processes, it is important to examine the neural manifestation of L2 novel word learning during the construction of a semantic network. Cognitive neuroscience technology of ERPs (Event-Related Potentials) is an ideal method because its high temporal resolution and specific neural activation component (N400) can be used to investigate the temporal neural activity during L2 new word learning. N400 effect (Kutas and Hillyard, 1980) is generated by the “semantic violation” paradigm that deliberatively replaces a word in a sentence with another word that is in conflict with the sentence, e.g., “I see a person riding a cake…” The N400 effect refers to a negative-going effect in the ERP component in the sense that the target word (semantically incongruous) generated lower potential in the ERP waveform within the 300 ms-500 ms time window after the presentation of the target word. The effect is usually maximum in the central parietal area (Kutas and Federmeier, 2011).

## Background literature

### Episodic memory and semantic memory

Episodic memory and semantic memory constitute two major categories in the long-term memory system (Kormi-Nouri et al., 2003; Moscovitch et al., 2006; Staudigl and Hanslmayr, 2013). In terms of context dependence, episodic memory involves the recollection of the original contexts in which the events occur, while semantic memory is mostly context-free (Squire and Zola, 1998; Elward and Vargha-Khadem, 2018). Newly-established semantic representations can be detached from original contexts and be abstracted as an independent entity to be flexibly generalized to other contexts (Hannula and Greene, 2012). As such, episodic memory and semantic memory must have different learning and forgetting patterns, and a comprehensive understanding of L2 new word learning should entail an examination of both processes. The difference has motivated neuroscientists to identify the neural foundations underlying their difference. Various neuroimaging methods have been employed to study the neural mechanisms of encoding and retrieving episodic memory and semantic memory (Tulving et al., 1994; Kraut et al., 2004; Wimber et al., 2009; Binder and Desai, 2011), and many studies have provided neuroscience evidence supporting the distinction between episodic memory and semantic memory in neural anatomical features (Cabeza and Nyberg, 2000; Opitz, 2010; Battaglia et al., 2012; Hirni et al., 2013). Aside from the neural anatomical differences, there was an alternative opinion proposing that the difference between episodic memory and semantic memory may lie in the neural dynamic patterns. This was supported by the significant overlap in the brain regions associated with episodic and semantic memory (Nyberg et al., 2002, 2003; Rajah and McIntosh, 2005; Binder and Desai, 2011; Renoult et al., 2015).

### Taxonomic relation and thematic relation in the semantic network

The associations between novel words and existing concepts can be built through a variety of semantic relations, mainly including thematic relations and taxonomic relations (Mirman and Graziano, 2012), which are two important semantic components stored in the semantic memory (Borghi and Caramelli, 2003; Estes et al., 2011; Yee et al., 2018). Taxonomic relation organizes concepts based on the similarity of features or properties among concepts (Rogers and McClelland, 2004). For example, cow and sheep are connected through a taxonomic relation. Such taxonomic relations have supporting neural substrates in the brain. Researchers found that some patients with specific brain injuries have difficulty in processing either living objects or inanimate objects (Warrington and McCarthy, 1983), which demonstrated neural anatomical specificity of taxonomic relations in the semantic memory system. Thematic relation refers to the relations between different concepts that usually co-occur or are involved in the same situation or event. For example, cow and grass, sheep and grass, are connected through a thematic relation. The existence of thematic relation in the semantic network has been commonly supported by a priming paradigm that found a stable priming effect between thematically related pairs of words (e.g., Hare et al., 2009). The processing of taxonomic relations and thematic relations has been found to involve different neural mechanisms and brain regions (Estes et al., 2011; Schwartz et al., 2011; Lei et al., 2020). Yet, they are not completely isolated, but are rather coordinated, allowing switching between each other (Landrigan and Mirman, 2018). In accordance with the property of different types of semantic relations, in the testing session of the current research, we designed the new discourses providing new contexts or new category features to examine whether the concepts’ integration via the thematic relations and taxonomic relations can be realized.

In studying the semantic network, many researchers focused on the mother tongue. For example, in the experiment conducted by Zhang et al. (2019), both taxonomic relations and thematic relations can be established in the process of concept acquisition in L1. We argue that studying L2 learning would give us different insights into language learning. The differences between L2 and L1 in the semantic networking patterns have been reported. Malt and Sloman (2003) concluded that the semantic categorization of concrete objects (e.g., bottles and dishes) is never completely established in L2. However, Li et al. (2011) reported that Chinese bilingual adolescents have the same awareness of the taxonomic relations both in L1 and L2, but the thematic relations in L2 need more cognitive resources to comprehend than taxonomic relations. There is also evidence supporting that not all kinds of conceptual representations are accessible in L2 to the same extent as in L1 (Nanjappa et al., 2016). Therefore, to provide more results in the research of L2-based semantic network establishment to be compared with that previous research based on mother tongue, in the present study, the scope was set to be in the study of semantic network establishment in L2.

### Multi-contextual word learning and its importance

In the process of novel concepts being integrated into the conceptual semantic network, multi-contextual learning plays a crucial role (Johns et al., 2016; Van den Broek et al., 2018). By means of exposing learners to abundant linguistic contexts, the lexicon information from unfamiliar inputs would be collected and stored in the lexical network in a more effective way (Saffran et al., 1997; Coutanche and Thompson-Schill, 2014). Based on the ERP method, many researchers used the contextual learning paradigm to study word learning (Mestres-Missé et al., 2007; Batterink and Neville, 2011; Zhang et al., 2019) and proved that learners can acquire novel concepts solely from the contexts. In restricted discourses, when specific information is provided, language learners can make inferences about the meanings of unknown words (Frishkoff et al., 2010; Borovsky et al., 2012, 2013) even when there may be only one context in which the novel word appears (Borovsky et al., 2010, 2012). However, under this circumstance, the lexicon knowledge that is acquired by learners is usually not complete and robust. This means that learners may only acquire part of words’ meaning which are susceptible to variation when more contexts are involved. Thus multi-contexts are required to establish robust and stable lexico-semantic representations. Furthermore, making novel words appear in several different contexts is more beneficial to learners’ intensive lexico-semantic learning than repeatedly showing the word in the same context (Bolger et al., 2008). Therefore, the diversity of contexts is very important for learners to gain a more stable and richer semantic representation (Rodríguez-Fornells et al., 2009; Beck et al., 2013).

## Research gap

Most of the previous experimental studies on word learning mainly used the approach of creating pseudowords from existing concepts (Mestres-Missé et al., 2007). This approach examines the process of associating novel word forms with known concepts rather than unknown concepts. Although adults’ word learning process mostly involves the refinement of known concepts, the acquisition of unknown concepts is still a very important part, the mechanism of which is less attended in the literature and is explored in this study.

Previous studies have mostly focused on the process of integrating novel words into the episodic memory and semantic memory in L1 (Ding et al., 2017a,b), and the language tasks that have been adopted are always based on the native language of participants. In comparison with those studies, less attention has been paid to how novel concepts are integrated into the existing L2 semantic network through the establishment of taxonomic relations and thematic relations. To fill this gap, the current study adopted L2 materials in the whole experiment and examined the acquisition processes of L2 novel words from multiple L2 episodic contexts.

Most previous studies adopted the semantic priming paradigm to study novel word learning in which the priming effect was elicited by word pairs (e.g., Zhang et al., 2019). The semantic priming paradigm is a suitable paradigm for probing word processing and associated learning effects because it is a well-controlled paradigm. However, in the current study, we adopted a naturalistic reading paradigm in which participants were required to read discourses or sentences embedded with the target words and process the context-word congruency. The reason that we adopted a discourse/sentence reading paradigm is that we attempted to create a text-processing activity that is closer to a naturalistic situation, thus enhancing the study’s ecological validity. In our daily reading, words mostly appear in a way that is embedded into various contexts (discourses, sentences, phrases). Word recognition is realized by combining background information from contents preceding the target word, across multiple time scales. Therefore, the discourse/sentence reading paradigm is more similar to such a naturalistic reading process in real life.

## The current study

The aim of this study is to examine the processes of learning novel concepts in L2 through the building-up of taxonomic relations and thematic relations in the conceptual semantic network, and how these processes depend on the memory types being acquired, i.e., episodic memory and semantic memory. The specific questions are formulated below.

Question 1: During multi-contextual learning, can novel concepts in L2 learned from episodic traces form new episodic memory? And how are relevant processes manifested in neural activity?

It is hypothesized that novel words during L2 learning can enter the episodic memory through the multiple episodic traces provided in contextual learning, which would be reflected in the larger N400 effect (the larger amplitudes obtained by substracting congruous condition from incongruous condition) elicited in the processing of recurrent discourses.

Question 2: During multi-contextual learning, can novel concepts in L2 be integrated into the semantic memory and conceptual semantic network by establishing new thematic relations and taxonomic relations? And how are relevant processes manifested in neural activity?

It is hypothesized that novel words during L2 learning can enter the semantic memory through the establishment of thematic relations and taxonomic relations, which would be reflected in the N400 effect elicited in new discourses or sentences. However, the relative strength of recurrence discourses, new-theme discourses, and category-feature discourses, i.e., the relative contribution of episodic memory and semantic memory (thematic relations and taxonomic relations) in the process of acquiring novel concepts is not included in the scope for the current research.

## Methods

### Participants

All participants provided written informed consent before taking part in this study and were paid the participation fee. There were 88 adults (18–25 years old with an average age of 20.7 years old, 46 females) who participated in the formal experiment. The chance level accuracy of the task in this experiment in the test session is 50% because the answer to each discourse is either true or false. One male participant did not reach 50% accuracy and was thus removed from the analysis, leaving 87 valid data samples. Participants were all right-handed and had the normal or corrected-to-normal vision and normal hearing. They were all native Chinese speakers. All of them were healthy and had no known (psychological, neurological, auditory, or linguistic) disorders. They had English as their L2 (excluding dialects) and can comprehend written English well. All participants passed the college-entrance English examination or the postgraduate-entrance English examination. The majority of Chinese students learned English as a foreign language as required in the education system but not for daily use purposes. Therefore, their English proficiency is at a relatively lower level as compared to the native level. The participants in this study never participated in a similar experiment before. All of them were undergraduate students or postgraduate students at Shandong Normal University. Ethical approval for this study had been obtained from the Human Research Ethics Committee (The University of Hong Kong).

### Learning session

#### Materials

The task program (including the learning session and the testing session) was designed and carried out by Psychtoolbox-3 in Matlab. In the learning session, 6 discourses embedded with 2 novel words were learned in the practice part, and 60 discourses embedded with 20 novel words were learned in the formal part. All learning materials were written in English. Each novel word is embedded into three discourses that represent three different contexts or themes. The novel word represents items that are probable to appear in these contexts. Each discourse describes one theme and is composed of one to two sentences. There is only one novel word to be learned in each discourse and the novel word only appears once in each discourse. Participants were notified that in the testing session they will be tested on the meaning of the words that they have learned in a certain way, but they were not told the detailed testing procedure. An example of a novel word and its discourses are shown below:

The novel word: *speth.*

Learning discourses:

1. *John is a construction worker, and he has to eat outdoors in winter. To keep his fingers warm, he turns on the heating button on the speth.*
2. *John’s child is 5 years old, and he no longer needs to use the spoon when eating. As a Chinese, his child has learned to pick up food using the speth.*
3. *John is making the dessert. According to the recipe, firstly, he should add the flour to the eggs, and mix them well using the speth.*

Designed meaning of “speth”: *chopsticks that can heat themselves.*

##### Novel words

Twenty-three nonderivational pseudowords were used as novel words in the discourses. All of these pseudowords were selected from the experimental materials used in the work of Deacon et al. (2004). According to this research, the pseudowords conform to the spelling and pronunciation rules of English but are not derived from known words. These pseudowords cannot be easily inferred from known words. On average, the length of these pseudowords is 4 to 6 letters long.

In this study, all pseudowords were assigned with a created definition. These definitions are based on people’s existing world knowledge but with slight twisting. They are not peculiar concepts and can be understood by participants. The definitions indicated the categories to which the novel concepts belong, as well as one to two additional features or functions that are slightly inconsistent with the categories, for example, “the duck that can fly and likes eating chili.” Therefore, these definitions can be regarded as describing a novel concept belonging to a novel subcategory. These explicit definitions were not presented to participants during the learning session and were supposed to be inferred by participants. All of these pseudowords are nouns and they play a concrete role in the discourses. They all appeared at the final position of the corresponding discourses. The reason to place them at the end is to enable the design of the N400 paradigm in which the processing of congruency of a word requires background context.

##### Discourses

The discourses should be restrictive in order for participants to infer the meaning of the novel words. Fifteen participants who did not take part in the main study were recruited to infer the meaning or definition of novel words. Here, the definition of each novel word is marked by several bullet points manifesting major features or categories of the word, which can be inferred from reading and correctly understanding the discourses. Participants were asked to read the discourses and write down the meaning of novel words, or the bullet points which constituted the definition of the novel word as much as possible according to their understanding. After that, we evaluated the answers that they wrote down by calculating the number of bullet points included in their answers. Ten pilot participants correctly inferred more than 80% of novel word meanings from the contexts The 5 pilot participants who did not pass 80% accuracy produced the scores as follows: 78.87, 77.46, 76.06, 76.06, and 42.25%. In addition, to provide participants with different exposure to the novel words, we ensured the variety of episodic contexts in the discourses. Ten participants who did not participate in the experiment were required to judge the contextual status of all sets of discourses (three discourses form a set for a novel word). Seven participants recognized the contexts as being different from each other for all sets of learning discourses. The length of the discourses is balanced.

Five sets of discourses (including 15 discourses with 5 novel words embedded) were grouped into one block. After the presentation of each block of discourses, there was a phase in which two contextual phrases were shown to the participants who were required to choose the one that is thematically related to the corresponding novel word that they just learned. One of the two options for each question is thematically-related phrases which include several keywords extracted from the discourses and illustrate the same context as that described in the discourses. These phrases are not highly repetitive of the materials in the learned discourses. The other option contains completely unrelated words or phrases. Each two-choice question corresponds to one of the three discourses for each novel word, so there are three two-choice questions for each novel word. In the practice session, there are 6 two-choice questions for 6 discourses with 2 novel words embedded; in the formal session, there are 60 two-choice questions, separated into four blocks with each block including 15 questions for 5 novel words. Another 20 participants were asked to use the 7-point Likert scale to assess the thematic relatedness between the discourses that are used as the learning materials and the related/unrelated phrases that are used in the two-choice questions. All participants judged that the related phrases were highly related to discourses and that the unrelated phrases were unrelated to discourses. The difficulty of the two-choice questions is relatively low, so in the formal session, repetition was quite rare. The average correct rate for the first round was 95.84%. For each participant, the average number of repetitions per choice is 0.05. An example of a binary choice question is shown below:

*Which one of the following two options, A and B, is thematically related to the novel word speth you just learned?*

*A: speth—Chinese way of picking up food.*

*B: speth—open the curtain after getting up.*

*Correct answer: A.*

#### Learning procedure

**Figure 1:**
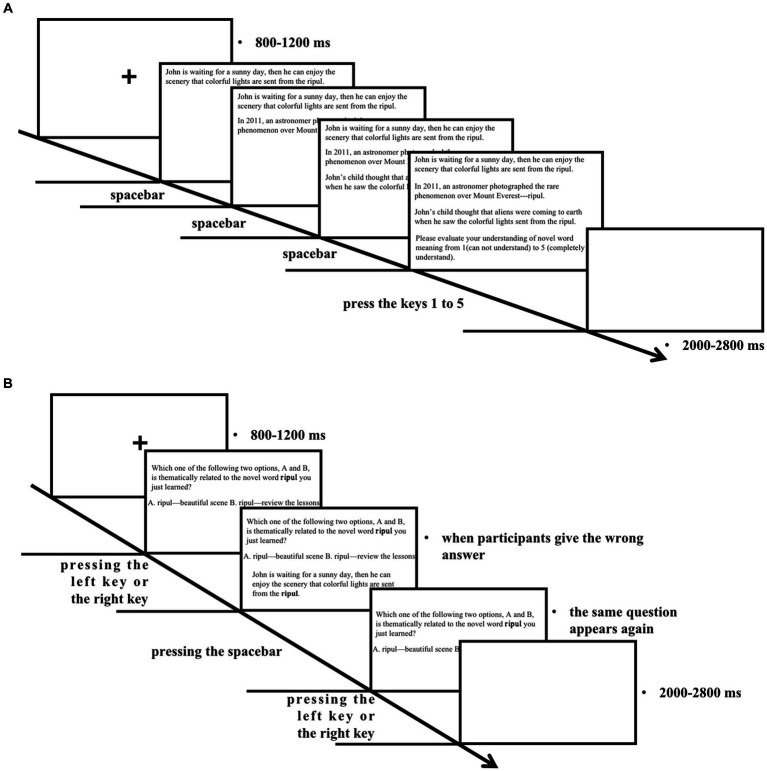
**(A)** Schematic overview of the learning session (learning discourses or sentences). **(B)** Schematic overview of the learning session (choice questions).

The basic procedures for the learning session are shown below (Figures 1A,B). Participants performed a self-paced reading task in a dimly lit, sound-attenuated computer lab. The viewing distance between the screen and the participants is around 60 cm. The words were set to Times New Roman font with a font size of 24 in sentences and the font size of 36 in single phrases. They are displayed in white color on a black screen on a 13-inch monitor. First, a fixation cross is displayed in the center for 800 to 1,200 ms (equally distributed, to avoid timing-dependent oscillatory EEG patterns). After that, three discourses for a single novel word are presented one by one and then presented together. After reading the three discourses, participants self-rated their understanding of all discourses on a 5-point scale (one represents “cannot understand” and five represents “completely understand”) by pressing the number keys. The participants’ ratings for these discourses were relatively high (*M* = 3.87, SD = 0.53), which shows that the difficulty of these discourses is at a proper level for participants to understand. After the response, a blank screen appears with a duration equally distributed from 2000 to 2,800 ms prior to the start of the next trial. The participants can take a short break after learning every five novel words. The presentation order of discourses within the same set and the same block was randomized across all participants. The presentation order of the blocks was also randomized.

As mentioned before, for every 15 discourses (5 novel words), there are 15 two-choice questions for participants to answer. Feedback on correctness was given to participants. The correct answer will not be on the same side three consecutive times. If participants give a wrong answer, the corresponding discourse will be presented once again for participants to learn, followed by a re-test. This will be repeated until participants give the right answer, which will lead to the next question after a duration equally distributed between 2000 ms and 2,800 ms. The presentation order of the 15 questions for every 15 discourses was randomized.

### Testing session

#### Materials

The testing session was conducted right after the participants were mounted with an EEG cap after the learning session. In the practice session, there are in total 10 discourses or sentences embedded with two novel words. In the formal session, there are a hundred discourses or sentences embedded with 20 novel words. All testing materials are written in English. There are in total three types of discourses or sentences in the testing session: 60 recurrence type discourses, 20 new-theme type discourses, and 20 category-feature sentences. In the testing session, all of the novel words were strictly placed at the final position of the discourses or sentences. That means that the participants do not only process the familiarity of the discourses and sentences but also need to judge whether the word that appeared at the final position matches the discourses/sentences. In recurrence type discourses, each of the 20 novel words appeared three times. In new-theme type discourses and category-feature sentences, each of the 20 novel words appeared once. Each type of discourses or sentences are divided into congruous and incongruous ones. In incongruous discourses or sentences, the incongruous novel words are selected from other novel words that were designed to match other discourses in the learning session.

##### Recurrence type

The discourses that participants have learned in the learning session and were used in the testing session are termed the recurrence type discourses. Half of the recurrence type discourses are totally the same as the learning session, and thus are congruous; the other half of recurrence type discourses were changed by replacing the original novel words with unmatched newly-learned words in the learning session, thus incongruous. The pairing of novel words and discourses in the incongruous recurrence type is pseudo-random. The processing of the novel words in the recurrence type is assumed to strongly depend on episodic memory since it involved the recollection and recognition of original contexts. An example of a recurrence type discourse is shown below:

*John made a wish to be given some good luck, hoping that in the sky he can have the chance to see the slethy.*

*Answer: false.*

##### New-theme type

The establishment of a new and independent thematic relation in the semantic memory concerning a novel word has to be tested by changing the context in which the word first appeared. Therefore, we introduced the new-theme type of discourses in the testing session that describes new themes in which the novel words are likely to occur. In these new-theme discourses, half were designed to be congruous and half incongruous. The length of discourses of this type is made similar to the recurrence type. It is worth noting that the new-theme discourses are totally new discourses that describe totally new contexts that have not been exposed to participants before. The similarity between new-theme discourses and learning discourses is very low.

Fifteen participants who did not take part in the experiment evaluated whether the new-theme discourses describe different themes from that learned in the learning session, and 11 of them confirmed that more than 73% of new-theme discourses described different themes from those described in the learning discourses. Fifteen participants who did not take part in the experiment were asked to infer the meaning or definition of the novel words from the new-theme discourses. They were asked to read the new-theme discourses and write down the meaning of the novel words, or the bullet points which constituted the definition of the novel word as much as possible according to their understanding. We marked the bullet points of novel words’ meaning which can be inferred from the new-theme discourses, and calculated the number of bullet points included in each participant’s answer. Ten of them correctly answered more than 80% of bullet points, and the 5 participants who did not pass 80% accuracy produced the scores as follows: 77.78, 71.11, 71.11, 68.89, 44.44%. An example of a new-theme type discourse is shown below:

*John was eating lunch. The food dropped and dirtied John’s new T-shirt when he tried to pick up food using the over-heated speth.*

*Answer: true.*

##### Category-feature type

To examine the learning effect of the novel words in terms of forming new taxonomic relations, in the testing session, we also introduced sentences describing categories and features of the novel words. Again, half of the category-feature sentences are congruous and the other half are incongruous. The incongruency was implemented by replacing the congruent one with another word from the list of the newly-learned words. Category-feature sentences are also totally new sentences, which have never been exposed to the participants before. The similarity between the category-feature sentences and learning discourses in the learning session is very low. An example of a category-feature sentence is shown below:

*The chopsticks that can heat themselves are speth.*

*Answer: true.*

Because the discourses or sentences used in the new-theme and category-feature conditions are new and dissimilar to those shown in the learning session, it is assumed that the word processing and recognition in these two conditions are not supported by episodic memory.

#### Testing procedure

**Figure 2:**
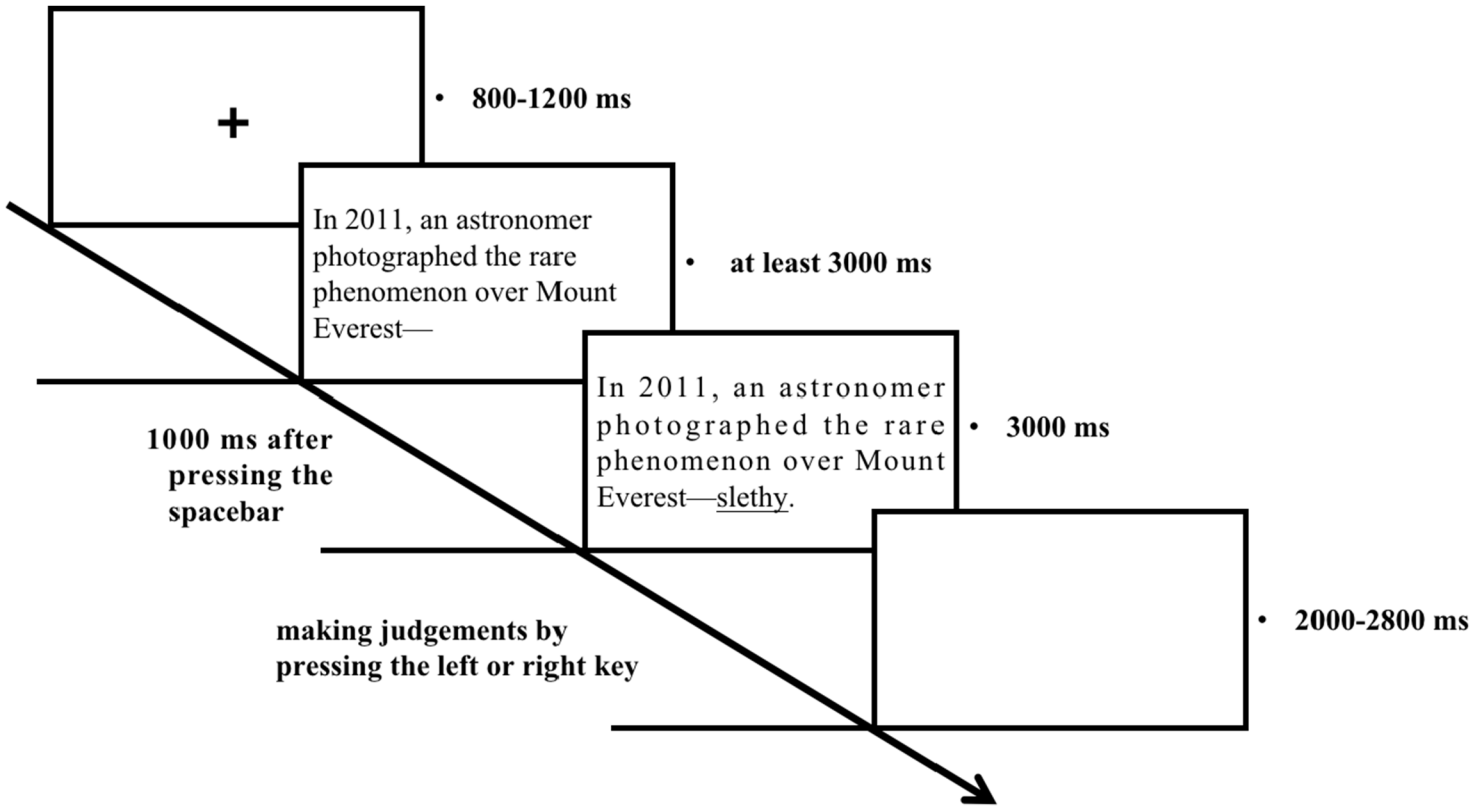
Schematic overview of the testing session.

The basic procedures for the testing session are shown below (Figure 2). First, a fixation cross appeared in the center of the screen with a duration of 800 to 1,200 ms (equally distributed). The discourses or sentences are presented one by one. The whole discourse except for the novel word is presented on the screen for participants to understand. When participants finish reading it, they press the spacebar and the novel word appears after 1 sec. Then the whole discourse stays on the screen for 3 sec for participants to judge whether the discourse is congruous or not by pressing the left (congruous) or right key (incongruous). After that, a blank screen appears with a duration of 2000 to 2,800 ms (equally distributed). Participants were asked to make judgments as rapidly and correctly as possible. The program continues if there is no response within 3 sec. Discourses containing the same novel words or describing the contexts for the same novel word were separated by other discourses.

In the testing session, the presentation order of the 100 discourses is as follows: 20 recurrence discourses---20 new-theme discourses---20 recurrence discourses---20 category-feature sentences---20 recurrence discourses. In other words, the 100 discourses or sentences were divided into 5 blocks. Within each block, the presentation order of discourses or sentences is randomized. The purpose of alternating the three types is to reduce fatigue. In the whole testing session, participants were instructed to keep still and try to avoid blinking during the judgment process. Participants were required to take a short break after every 10 trials.

## EEG data collection and preprocessing

The brain electrical activities were recorded with 64 Ag/AgCl electrodes (Brain Products, Germany) on an electrode cap according to the international 10–20 system. The data were sampled at 1000 Hz and later resampled to 250 Hz for offline prepossessing. The ground electrode was placed between FP1 and FP2. FCz was used as the physical reference during the EEG collection and the average reference was used in offline processing. The electrode impedance was kept below 5 kΩ throughout the experiment. Letswave 7 (André Mouraux, Brussels, Belgium) and EEGLAB (Delorme and Makeig, 2004) were used to preprocess and analyze EEG data. The band-pass filter was applied in the range of 0.05–30 Hz and EEG artifacts were removed by the SPA toolbox (Ouyang et al., 2022). SPA is a simple yet highly cost-efficient method that has been validated in its ability to extract ERP and preserve ERP effects, which is based on the principle of removing unnecessary components instead of the whole trial while eliminating artifacts. Therefore, after the SPA, no trial is totally deleted from the dataset. Epochs of 1,200 ms were created relative to the onset of the last (novel) word in each discourse (200 ms before and 1,000 ms after the onset of stimulus) for generating ERPs baselined at a pre-stimulus interval of [−200 ms, 0 ms]. The ERPs amplitudes from specific time windows and electrodes were calculated and compared across conditions.

## Data analysis

Accuracy and reaction time were calculated and served as the major behavioral data in this study. The average accuracy, reaction time, standard deviation (SD) were reported first. Then, for each participant, we seperately calculated the accuracy and reaction time data under the congruous condition and the incongruous condition from three types of discourses and sentences, and paired ttests were conducted to examine whether there were significant differences between congruous and incongruous conditions. ERPs amplitudes from specific time windows and electrodes (reported in the Results section) were calculated as the neural data. For statistical testing, repeated measures ANOVA and paired t-tests were conducted to test the statistical significance of the neural variable between conditions. T value, *p* value, degree of freedom, SEM (standard error of the mean), and effect size measures were reported. Multiple comparisons correction based on Bonferroni correction were conducted wherever needed (Shaffer, 1995). The outliers (beyond mean ± 3 SD) from the behavioral analysis (0.25% excluded) and neural analysis (0.82% excluded) were excluded.

In this research, the effect was mainly manifested in the neural data rather than the behavioral data. We hypothesized that the semantic processing of the novel words occurs in the brain, regardless of whether participants gave the wrong or right answer. In this sense, the relevant neural effect of semantic processing at single trials was treated as a continuous variable. Below a threshold at this continuous variable, the participant may generate a wrong answer. However, we have to include all the trials to have all the neural effects (no matter to what degree) retained in our statistical analysis to reveal the neural effects.

## Results

### Behavioral measurements

#### Accuracy

The average accuracy, SD, and CV of all participants are reported in Table 1 separately for all discourses combined, different discourse types, and different blocks of the recurrence type. The average accuracy is relatively high (substantially higher than 50%) and the cross-individual variability (SD and CV) is relatively low, which reflects behavioral validity. For the multiple t-tests, we adopted the Bonferroni correction to adjust the significance level α. For the t-test results for all discourses combined (the entire dataset), we retain the original standard significance level (α = 0.05) because testing on the whole dataset of the entire study can be treated as a single test. Then, when analyzing (testing) the three types (recurrence type, new-theme type, category-feature type), they are treated as three tests. Therefore, based on Bonferroni correction, the significant level α is adjusted by dividing the original value by three. Thus, the new α to be compared with is 0.017. Since the recurrence type is further divided into three blocks, the new α value for the further divided subsets becomes 0.006, i.e., *p* values less than 0.006 (rather than 0.05) will be treated as significant results. As shown in Table 2, from the t-test results of accuracy, it is found that new-theme type, category-feature type, and the first and the third block of recurrence type elicited the statistically significant congruency effect.

**Table 1:** The accuracy data for all discourses combined and different types or blocks of discourses.

Accuracy	Mean	SD	CV
Overall	0.79	0.13	0.16
Recurrence type	0.80	0.13	0.17
New-theme type	0.76	0.15	0.20
Category-feature type	0.81	0.14	0.18
Recurrence type block 1	0.77	0.14	0.19
Recurrence type block 2	0.80	0.17	0.21
Recurrence type block 3	0.82	0.13	0.16

SD refers to standard deviation. CV refers to the coefficient of variation.

**Table 2:** The congruency effect in accuracy data from all discourses combined, three types, and three blocks of recurrence type.

	*t* value	*p* value	Adjusted *α*	df	SEM	Cohen’s d
Overall	−1.86	0.07	0.05	86	0.02	0.16
Recurrence type	−0.532	0.60	0.017	86	0.02	0.04
New-theme type	−2.62	0.01	0.017	85	0.03	0.21
Category-feature type	−3.04	<0.01	0.017	85	0.02	0.29
Recurrence type block 1	−3.79	<0.01	0.006	84	0.02	0.37
Recurrence type block 2	0.66	0.51	0.006	84	0.02	0.05
Recurrence type block 3	4.96	<0.01	0.006	83	0.02	0.43

#### Reaction time

The average reaction time, SD, and CV of all participants are presented in Table 3 for all discourses combined, different discourse types, and different blocks of the recurrence type. The average reaction time is relatively short (between 1 and 1.3 s), and the cross-individual variability (SD and CV) is relatively low. This indicates that the tasks were not so difficult that would require the participants to undergo complex and deliberate mental processes to complete each trial that may last for seconds, in which case the ERPs paradigm would not be suitable because ERPs usually capture sub-second neural cognitive activity. From the results of reaction time (Table 4), it is found that the data under all conditions, except the third block of recurrence discourses, elicited a significant congruency effect. It can be noticed that the behavioral response (reaction time and accuracy) for category-feature sentences has a substantial difference between congruous condition and incongruous condition, which was not observed in the neural level (see below).

**Table 3:** The reaction time data for all discourses combined and different types or blocks of discourses.

Reaction time	Mean (ms)	SD	CV
Overall	1,182	0.26	0.22
Recurrence type	1,161	0.26	0.22
New-theme type	1,255	0.29	0.23
Category-feature type	1,161	0.28	0.24
Recurrence type block 1	1,298	0.34	0.26
Recurrence type block 2	1,150	0.28	0.24
Recurrence type block 3	1,036	0.25	0.24

SD refers to standard deviation. CV refers to the coefficient of variation.

**Table 4:** The congruency effect in reaction time data from all discourses combined, three types, and three blocks of recurrence type.

	*t* value	*p* value	Adjusted *α*	df	SEM	Cohen’s d
Overall	4.69	<0.01	0.05	86	0.01	0.16
Recurrence type	3.78	<0.01	0.017	86	0.01	0.15
New-theme type	2.41	0.02	0.017	85	0.02	0.13
Category-feature type	4.62	<0.01	0.017	86	0.02	0.21
Recurrence type block 1	4.48	<0.01	0.006	86	0.02	0.22
Recurrence type block 2	3.27	<0.01	0.006	86	0.02	0.15
Recurrence type block 3	−0.69	0.49	0.006	85	0.02	0.03

### Event-related potentials

**Figure 3:**
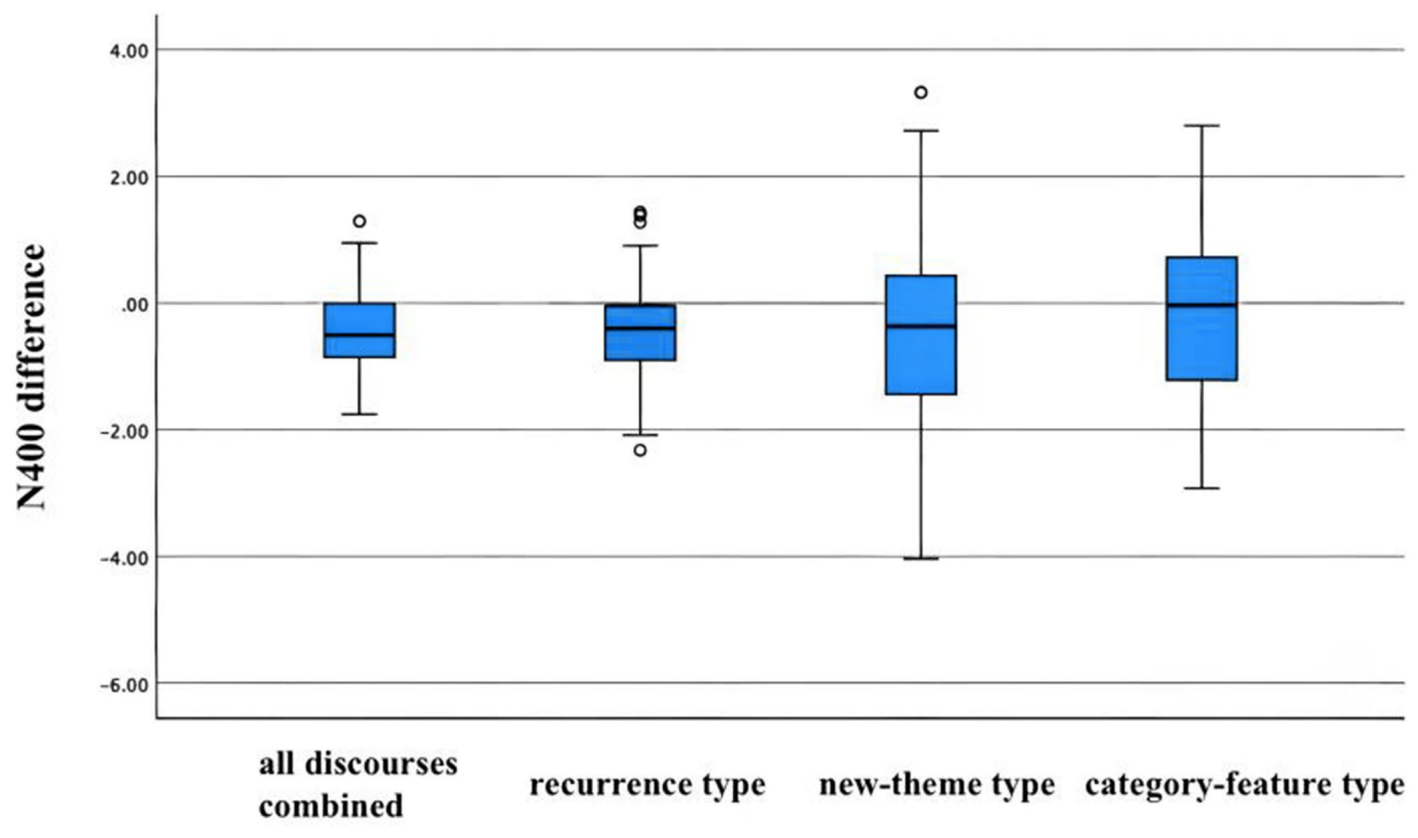
The boxplot displayed the difference amplitudes within the N400 window from various discourse conditions.

ERPs were analyzed to examine the effect of the congruency of the target word in a sentence on the amplitude of ERP in the N400 time window. The target words are the novel words learned by participants. The N400 effect is an indicator of the degree of semantic congruence of a word with respect to its context perceived by participants. It is also an indirect indicator of the participants’ learning performance because unrecognized words will not generate incongruency. Based on previous studies (Kutas and Hillyard, 1980; Mestres-Missé et al., 2007), the N400 effect was measured from the time window of 300–500 ms after the onset of the novel word. Here, the N400 effect refers to the difference in ERP amplitude between congruent and incongruent conditions in this time window. The representative electrodes Cz, CPz, and Pz are used as the region of interest (ROI) and in this research, they are averaged as a new virtual electrode for statistical analysis. This ROI of the central and parietal region is in line with many previous studies on the N400 effect (Mathalon et al., 2002; Hajra et al., 2018). To examine if the congruency effect (i.e., N400 effect) varies across different discourse types, we first conducted a repeated measures ANOVA with discourse type (recurrence type block 1, new-theme type, recurrence type block 2, category-feature type, recurrence type block 3) and congruence (congruous vs. incongruous) as two within-subject factors, and found a significant interaction effect between the two factors (*F* = 2.899, *p* = 0.022, Partial Eta Squared = 0.03). Besides, the main effects of both discourse type and congruence are significant [type: *F* (4,332) = 3.008, *p* = 0.018, Partial Eta squared = 0.04; congruence: *F* (1,83) = 33.649, *p* < 0.001, Partial Eta Squared = 0.29]. After the ANOVA test, paired t-tests were conducted on all discourses combined, the three types, and three blocks of recurrence type, separately. As shown in Table 5, for all discourses combined, there is a significant N400 effect. For separate types, the recurrent discourses elicited statistically a significant N400 effect; new-theme type discourses also elicited a statistically significant N400 effect; category-feature type sentences did not elicit a statistically significant N400 effect (see Table 5). Since the recurrent discourses are divided into three blocks, t-tests were also separately conducted on the three blocks of recurrence discourses (but the *p* values were compared to the Bonferroni-adjusted α level). The results showed that at the average site, the recurrence discourses in blocks 1 and 3 elicited significant N400 effects (see Table 5). The boxplot was used to display the difference amplitudes within the N400 window from various discourse conditions (Figure 3).

**Table 5:** The N400 effect of the target word from all discourses combined, three types, and three blocks of recurrence type at the average electrode.

Averaged electrode	*t* value	*p* value	Adjusted *α*	df	SEM	Cohen’s d
Overall	−5.83	<0.01	0.05	85	0.07	0.29
Recurrence type	−5.81	<0.01	0.017	85	0.08	0.32
New-theme type	−3.33	<0.01	0.017	86	0.15	0.24
Category-feature type	−0.60	0.55	0.017	86	0.15	0.05
Recurrence type block 1	−4.55	<0.01	0.006	85	0.15	0.36
Recurrence type block 2	−2.63	0.01	0.006	84	0.12	0.18
Recurrence type block 3	−2.87	<0.01	0.006	86	0.12	0.20

Df refers to the degree of freedom. Regarding the dfs for different types of discourses or sentences, the reason is that we separately examined the number of outliers in the data sample for different types (but based on the same criterion), and removed the different number of outliers in different types. Therefore, the actual numbers of participants that are used in the *t* test analyses for different types of discourses or sentences were different. SEM refers to the standard error of the mean.

**Figure 4:**
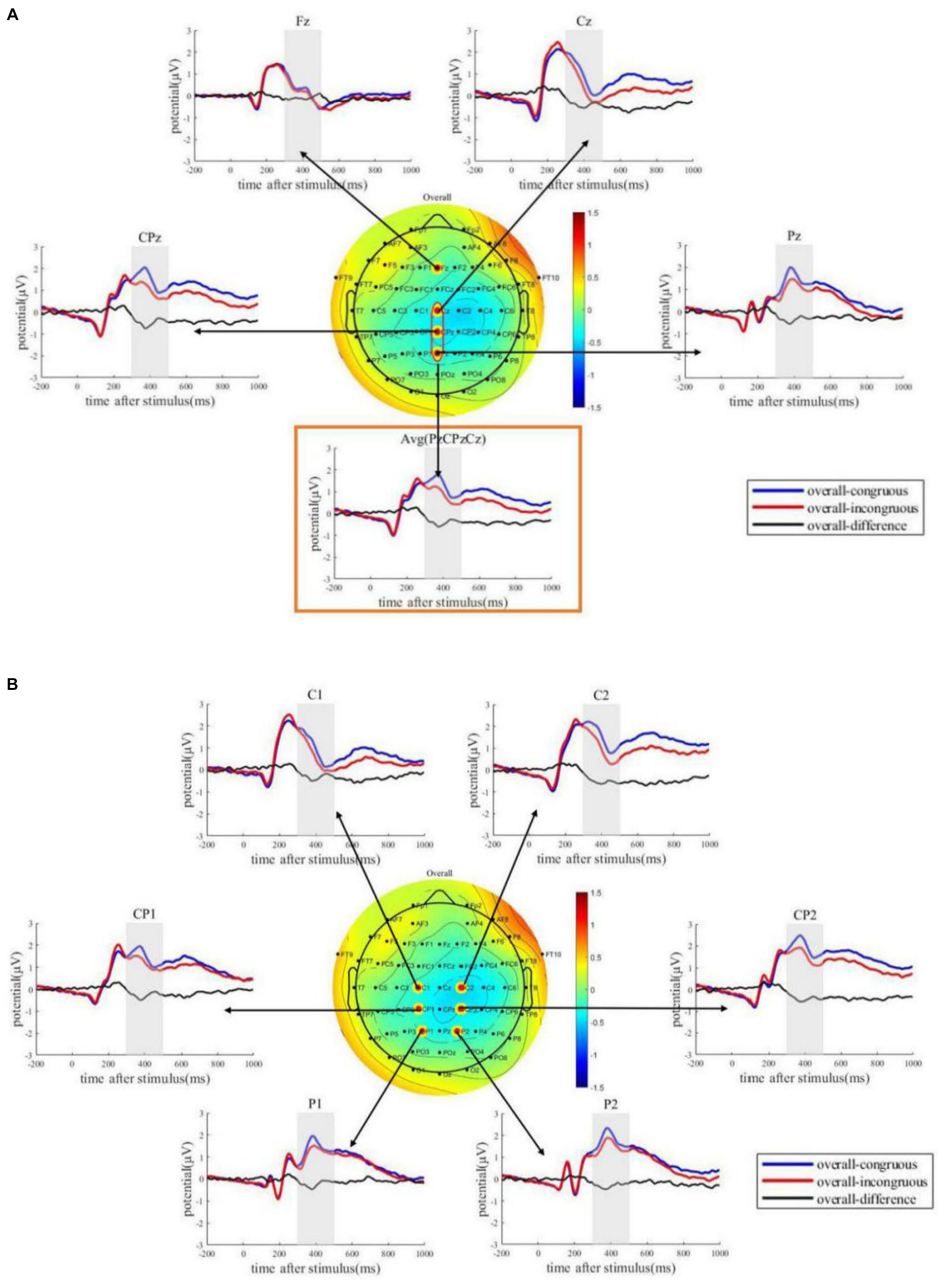
The overall pattern of N400 effects. **(A)** ERPs waveforms elicited by the novel words in the congruous discourses and incongruous discourses, as well as the ERPs differences from Fz, Cz, Pz, CPz, and the average electrode (averaged from Cz, Pz, and CPz). **(B)** ERPs waveforms elicited by the novel words in the congruous discourses and incongruous discourses, as well as the ERPs differences from C1, C2, P1, P2, CP1, and CP2. The topography shows the average ERPs amplitude differences within the time window of [300 ms, 500 ms].

The overall N400 effect averaged from 300 to 500 ms displays a typical central-parietal topography (Figure 4). From this central-parietal distribution, we selected a few electrodes to present the ERPs waveforms. The grand average ERPs waveforms elicited by the novel words (i.e., the last word in each discourse) for both congruous and incongruous conditions at the electrodes Fz, Cz, CPz, Pz, and the average electrode are presented in Figure 4A to show the waveform of midline ERPs. The waveforms at the electrodes CP1, CP2, C1, C2, P1, and P2 are presented in Figure 4B to show the waveform of ERPs surrounding the midline. Figure 4 shows that the ERPs waveforms clearly differ between congruous and incongruous conditions. The two ERPs start to diverge at around 300 ms after stimulus onset. The overall pattern aggravated by all conditions serves as a straightforward demonstration of the existence of learning effects. Next, we examined the difference in this learning effect across different conditions.

**Figure 5:**
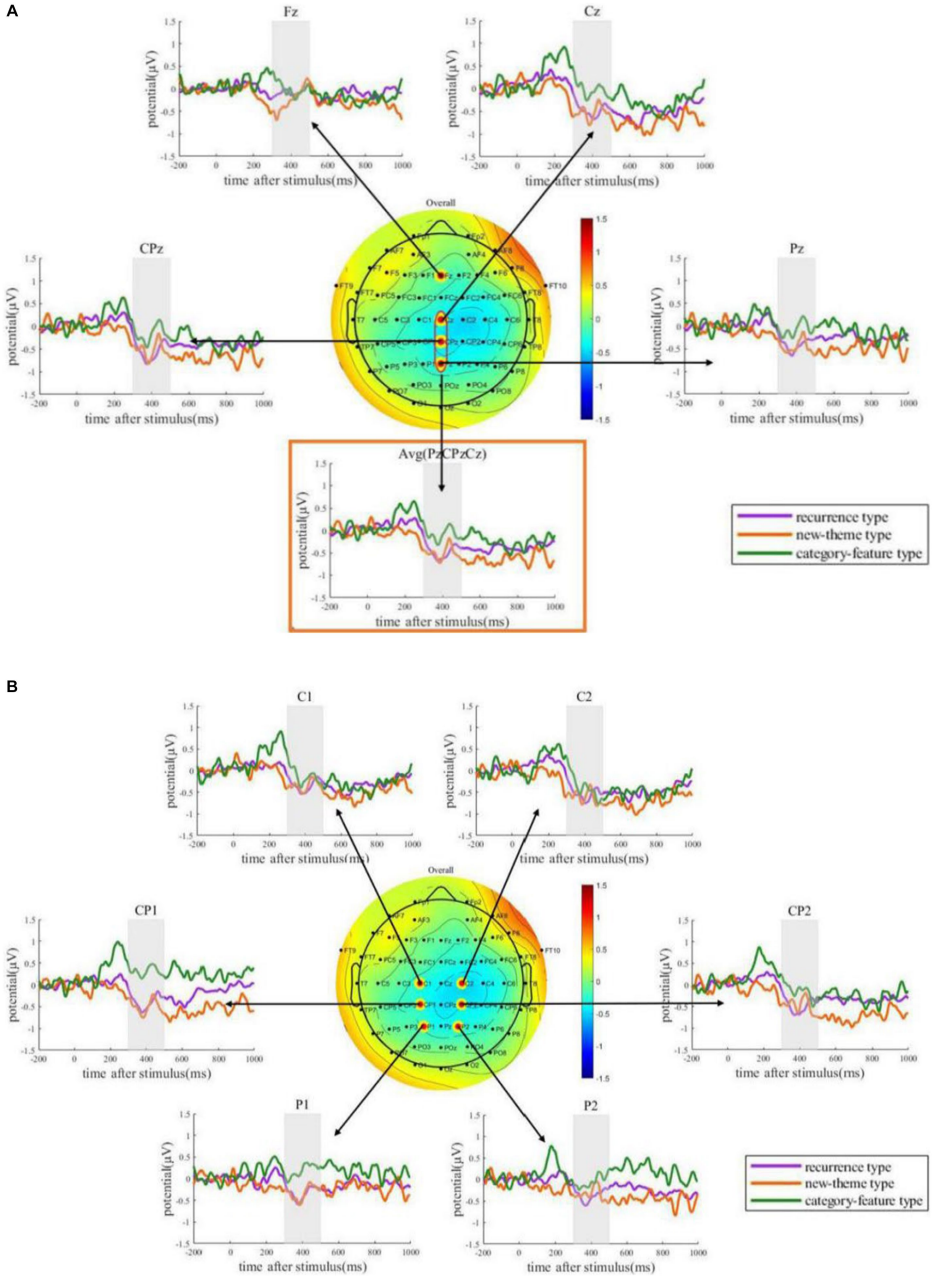
ERPs differences result from the three types: recurrence type, new-theme type, and category-feature type. **(A)** Waveforms for the N400 effects elicited by novel words in different types of discourses in midline area. **(B)** Waveforms for the N400 effects elicited by novel words in different types of discourses in central and parietal area.

The grand average ERPs difference waveforms elicited by the novel words for the three types (recurrence type; new-theme type; category-feature type) are plotted in Figure 5 for midline electrodes and the surroundings. From these ERPs waveforms, it can be observed that the new-theme type and the recurrence type appear to have stronger N400s as compared to the category-feature type. This cross-condition difference were tested, and the statistical results were shown in Table 5.

**Figure 6:**
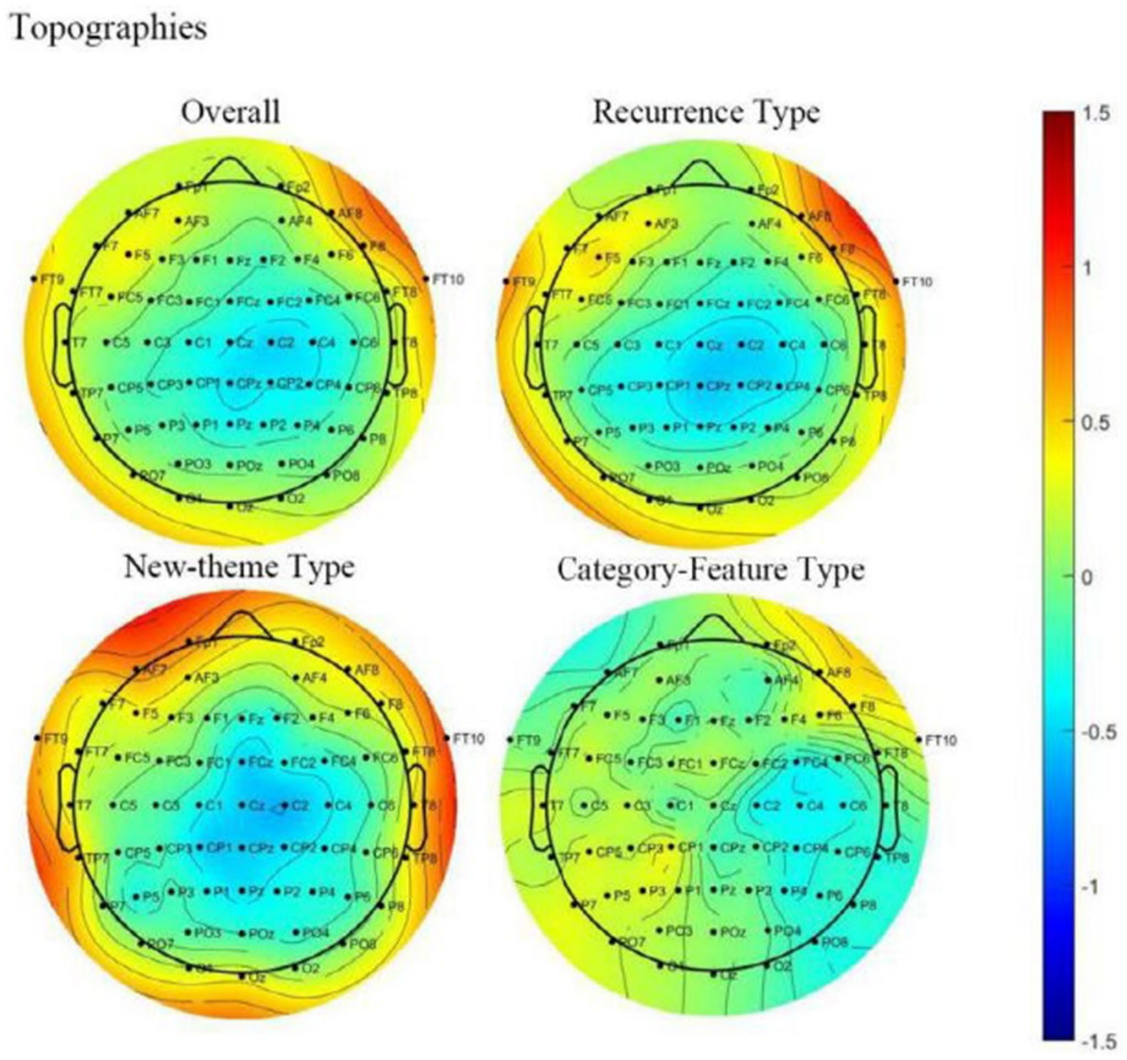
Topographies of ERPs differences between congruous and incongruous conditions for the overall data and different types of discourses averaged from a time window of [300 ms, 500 ms].

The topography pattern from Figures 4, 5 are both from the condition-average results. To show the difference in topography across different conditions, in Figure 6 we plotted the topography pattern for all data (overall), recurrence type, new-theme type, and category-feature type separately. The topography results show that there is a clear structure of the N400 pattern in all conditions except for the category-feature condition. The N400 topography pattern consistently showed a central-parietal distribution. The fact that a structured pattern is not observed in the category-feature conditions suggests that for the current study, a significant learning effect is not found in the taxonomic relation. The statistical results shown in Table 5 confirmed this: the N400 effects are significant in all conditions but the category-feature one.

## Discussion

This study employed ERPs technology to investigate the neural process of acquiring L2 unknown concepts from various episodic traces and integrating them into the episodic memory and semantic memory systems. Briefly, the ERPs results showed that the newly learned L2 novel words elicited significant N400 components by subtracting congruous contexts from incongruous contexts. This N400 effect was significant in the recurrent discourses, which answered the first question of this study, i.e., it indicated that novel words were integrated into the episodic memory through the multiple episodic traces learned in the contexts. Statistically, the N400 effect was significant in the new-theme type of discourses but was not significant in the category-feature type sentences, which partially answered the second question of this research, i.e., it indicated that novel words can be integrated into the semantic memory through the establishment of thematic relations. However, the establishment of taxonomic relations cannot be confirmed in the current study.

### Contribution of episodic memory to L2 word learning

In this study, the participants were presented with and tested on previously learned L2 words in a setting in which the words were presented under different L2 contexts (discourses) that are either congruous or incongruous with the word. The three contexts include (1) recurrence type: the discourse content in the testing session is the same as the learning session, (2) new-theme type: the discourse content in the testing session forms a new thematic relation with the tested word, and (3) category-feature type: the discourse content in the testing session forms a new taxonomic relation with the tested word. In the recurrence type, the processing of the novel L2 words was under identical contexts as in the learning session, thus the N400 effect should be primarily due to episodic memory. It has been proposed that, during learning processes, participants intend to infer the novel word meanings from the discrete episodes and personal experiences stored in the episodic memory (Moscovitch et al., 2006). Consequently, the inferred novel concepts with relevant episodic information are encoded in the episodic memory to be retrieved in the testing session, particularly when the testing context is identical to that in learning (Rugg et al., 2015). It has also been found that the performance of episodic memory in the process of retrieval is manifested as the recurrence of neural patterns in the hippocampus (Rugg et al., 2008). In this research, the various episodes that provided multiple contexts can further strengthen the formation of new episodic memory traces in the brain since the episodic memory system has always been regarded as a dynamic and context-dependent system (Staudigl and Hanslmayr, 2013). Enriched context facilitates the encoding of scattered and discrete episodic information for later retrieval. Taken together, it is expected that episodic memory contributes to new word learning, which is confirmed by the recurrent discourses in the testing session. However, whether such a process contributes to the semantic memory system cannot be known solely by the recurrence discourses. It has been claimed that encoding and retrieval from episodic memory contribute to the association of words and meaning in the semantic memory system (Greenberg and Verfaellie, 2010), thus episodic memory plays an important role in integrating novel concepts into the semantic memory system. Therefore, our experimental design introduced other types of discourses different from the recurrence type to examine how various episodic traces contribute to the integration of novel concepts into the semantic network, which is the core of word learning.

### N400 effects in the new-theme discourses confirm the formation of new semantic memory

As mentioned earlier, apart from episodic memory, concepts are organized into semantic memory via thematic relations and taxonomic relations. The statistically significant N400 effect in the new-theme discourses is the main novel part of this research because it demonstrated that, through multi-contextual L2 learning, the thematic relations that have been established for the unknown concepts of new words in the learning session have been successfully extended. This is unambiguously demonstrated by the significant N400 effect in the new-theme discourses that were not used in the learning session. This successful extension of thematic relations may be due to the contextual restriction and variability of the L2 learning contexts. Contextual restriction refers to the degree to which contexts restrict the clues provided for the meaning of novel words. The more restrictive the context is, the more likely the participants are to successfully establish the semantic representations (Borovsky et al., 2010). In principle, multiple restrictive contexts support the formation of valid thematic relations among novel concepts in the conceptual semantic network better than a single restrictive context. In addition to restriction, variability also plays a positive role as it would reinforce the understanding of new concepts. The effect of contextual variety on lexical meaning learning has been confirmed by many studies (Plummer et al., 2014; Johns et al., 2016; Temple et al., 2017). The shift from the learned themes to unlearned themes not only confirms an effective learning outcome of the new word but also marks a qualitative shift from episodic memory to semantic memory.

### N400 effects in the category-feature sentences show relatively weak semantic memory built in the taxonomic relation domain

The statistically non-significant result for the category-feature type sentences showed that, at least in the current study, we did not find strong evidence that the categories and features of the novel concepts have been successfully acquired by the participants. Therefore, the current study cannot confirm that the multi-context discourse, although effective in establishing and extending thematic relations, was also effective enough in building the taxonomic relation system for unknown concepts. This result is different from previous research which has shown that taxonomic relations are easier to acquire than thematic relations in the semantic network (Mirman and Graziano, 2012; Zhang et al., 2019). For this inconsistency, we proposed the following explanations.

First, previous studies on the conceptual semantic network of L1 or L2 mostly used known concepts linked to real words or pseudowords as experimental materials, whereas our study used unknown concepts in the form of L2 pseudowords. The process of learning unknown concepts is more of an exploration process, similar to the process of learning new concepts in childhood when the existing semantic network is not fully developed yet to efficiently cope with the unknown concepts. Previous developmental research on word learning has confirmed that concept learning develops with age (Sachs et al., 2008). Specifically, there is a transition of dominance from thematic relations to taxonomic relations across the development of an individual (Mirman et al., 2017). Learners with less developed semantic networks mainly acquire concepts through thematic relations since thematic relations involve richer contextual information and demand fewer cognitive resources (Lin and Murphy, 2001). In contrast, taxonomic relations are more abstract, requiring more cognitive resources and an efficient semantic network to generalize the core features of words (Blaye et al., 2006). Similarly, the participants in this study are L2 learners who can be considered to have a relatively underdeveloped semantic network since the overall L2 proficiency of the participants was relatively low as compared to the native level (Sheng et al., 2006; Wolter, 2006). Therefore, they may be constrained by the limited availability of cognitive resources, and thus may be more inclined to represent concepts in thematic relations. In contrast with unknown concepts, known concepts as used in previous studies, even when being shown in the form of pseudowords, have already established robust connections with other nodes in the semantic network including taxonomic relations.

Second, the distinction in the processing and performance of different semantic relations across different cultures may also provide an explanation for the results. Participants from eastern countries were shown to be better at making thematic judgments, while westerners tend to be better at making taxonomic judgments (Gutchess et al., 2010). Researchers believed that this may be related to the strategies that people adopt in the judgment of semantic relations implicated with cultural experience (Chiu, 1972; Ji et al., 2004; Unsworth et al., 2005). For participants who are cultivated in the individualist culture, their individual experiences facilitated them to perceive things in a logical and analytical way. However, for participants in the collectivist culture, their collectivist experiences promote them to perceive things in a holistic and rational way (Nisbett et al., 2001; Ji et al., 2004). Gutchess et al. (2006) found the neural basis for these cultural differences in an fMRI study: compared with Asian participants, American participants activated more brain regions related to object processing. The cultural difference may provide another possible explanation for the seemingly weak establishment of taxonomic relations in the current research.

Third, there may be a limitation in the current study which may lead to the result that we did not observe the significant N400 for the established taxonomic relations in the semantic network although this effect can be observed from the behavioral data. In the learning session, the design of choice questions may be a sub-optimal design. Participants may simply choose the alternative response option after failing in one attempt, which raises concern about the genuineness of the learning processes. It is nevertheless worth noting that the rate of making more than one attempt was very low (see above). Although the learning process is compromised to some degree, this limitation should not invalidate the ERP session because the N400 effect can only be elicited if the participant truly links the target word with the sentence context. In any case, the learning process should be improved in future studies using a similar design to enhance their learning. Improvement of learning quality may change the result regarding the category-feature sentences: the N400 effect in category-feature sentences may become significant if the learning design is improved. Before the testing session, we did not prepare the pretest for some extra participants to examine the nature of category-feature sentences, just as we did for new-theme discourses, which can be improved in future design. Overall, while it is true that more intense and quality learning and testing will eventually lead to a better neural outcome and thus a higher chance to see N400 effects in all conditions, here in the present work, we tried to present a scenario that the establishment of taxonomic relations appear to be more difficult than the other ones at the neural level.

### Innovative insights brought by the current study

To study the process of acquisition of new word meanings into learners’ memory systems, previous studies usually asked participants to infer known concepts in the form of L1 pseudowords by reading plenty of L1 materials, and the semantic priming paradigm is employed to evoke neural responses (Chen et al., 2014; Ding et al., 2017b). There are some innovative elements in the present study as compared to the previous ones. First, different from the semantic priming paradigm, the current study adopted the discourse/sentence reading paradigm which also proved to be an effective paradigm for studying the process of novel word learning, as shown by the significant neural effects associated with new word processing and recognition. Second, the current study totally adopted L2 materials, thus truly demonstrating relevant learning effects in L2. Third, the current study adopted a specifically-designed multi-contextual learning paradigm by which three different processes of memory pathways can be conveniently probed in the testing session, i.e., episodic memory pathway (by recurrent discourses) and two semantic memory pathways (by new-theme discourses and category-feature sentences). Fourth, by the design of multi-contextual learning in the learning session and sentence/discourse processing in the testing session, the current study made the entire learning and testing processes more naturalistic, which enhances the ecological validity of EEG-based study of new word learning in the field. Fifth, the aim of the current research is mainly to examine whether L2 concepts can be acquired via episodic memory and semantic memory. As an outlook for the future, we can apply some potentially more sensitive methods, such as MVPA (see Heikel et al., 2018; He et al., 2023) to further test if fine-grained differences between all types of memory formation processes may have differential impacts on semantic N400s.

### Regarding the ordering of different types of discourses

Here we also discuss another important point in this study: the arrangement of the presentation order for the three different types. We arranged the three blocks of recurrent discourses in a specific way to let them encompass the other two types (see Method). As the results showed, the first block and the third block of recurrent discourses elicited significant N400 effects. This is a demonstration that the existence of significant N400 effects is not systematically affected by the order. Most importantly, the reason that we did not observe a significant N400 effect in the category-feature type sentences is not simply due to the order effect (i.e., the fact that it is in the 4th block). We are making this clarification because one may question that the insignificance may be due to fatigue (in which case the effect should linearly decrease over the three recurrent blocks). This is ruled out by the fact that the 5th block, which is later than the 4th block (category-feature type), still shows a significant N400 effect. Accordingly, the fact that we did not observe a significant N400 effect in the category-feature type may imply a weaker learning outcome in the taxonomic domain as compared to thematic relation (new-theme type) and episodic-dominant learning (recurrence type).

## Conclusion

This study found that, for L2 adult learners whose language proficiency is relatively low compared with bilinguals, episodic knowledge played a significant role in the contextual learning of L2 unknown concepts. Importantly, the various episodic traces also enable novel concepts to be absorbed into the semantic memory by means of constructing extended thematic relations, but not taxonomic relations. For the taxonomic relations for novel concepts, we cannot conclude that they were established as the N400 effects were statistically insignificant (we also cannot conclude the other way around). However, future studies may help to better address this question by improving the learning quality. In sum, this research confirmed the contribution of contextual learning in new word learning in L2 learners based on neural evidence.

## Data availability statement

The original contributions presented in the study are publicly available. This data can be found at: https://osf.io/nfcrx/?view_only=383983a9c1374978a3d61d5e54690636.

## Ethics statement

The studies involving humans were approved by the Human Research Ethics Committee at the University of Hong Kong. The studies were conducted in accordance with the local legislation and institutional requirements. The participants provided their written informed consent to participate in this study.

## Author contributions

SX: Conceptualization, Data curation, Formal analysis, Investigation, Methodology, Resources, Software, Validation, Visualization, Writing – original draft, Writing – review & editing. HW: Data curation, Software, Writing – review & editing. SL: Data curation, Funding acquisition, Resources, Writing – review & editing. GO: Conceptualization, Formal analysis, Funding acquisition, Project administration, Resources, Software, Supervision, Validation, Writing – review & editing.
